# Coordinated Metabolic Changes and Modulation of Autophagy during Myogenesis

**DOI:** 10.3389/fphys.2016.00237

**Published:** 2016-06-16

**Authors:** Paola Fortini, Egidio Iorio, Eugenia Dogliotti, Ciro Isidoro

**Affiliations:** ^1^Department of Environment and Primary Prevention, Istituto Superiore di SanitàRome, Italy; ^2^Department of Cell Biology and Neurosciences, Istituto Superiore di SanitàRome, Italy; ^3^Università degli Studi del Piemonte Orientale “Amedeo Avogadro”Novara, Italy

**Keywords:** muscle differentiation, autophagy, metabolism, p53

## Abstract

Autophagy undergoes a fine tuning during tissue differentiation and organ remodeling in order to meet the dynamic changes in the metabolic needs. While the involvement of autophagy in the homeostasis of mature muscle tissues has been intensively studied, no study has so far addressed the regulation of autophagy in relation to the metabolic state during the myogenic differentiation. In our recently published study (Fortini et al., 2016) we investigated the metabolic profile and regulation of autophagy that accompany the differentiation process of mouse skeletal muscle satellite cells (MSC)-derived myoblasts into myotubes. Here, we briefly present these findings also in the light of similar studies conducted by other authors. We show that during myogenic differentiation mitochondrial function and activity are greatly increased, and the activation of autophagy accompanies the transition from myoblasts to myotube. Autophagy is mTORC1 inactivation-independent and, remarkably, is required to allow the myocyte fusion process, as shown by impaired cell fusion when the autophagic flux is inhibited either by genetic or drug manipulation. Further, we found that myoblasts derived from p53 null mice show defective terminal differentiation into myotubes and reduced activation of basal autophagy. Of note, glycolysis prevails and mitochondrial biogenesis is strongly impaired in p53-null myoblasts. Thus, autophagy, mitochondrial homeostasis, and differentiation are finely tuned in a coordinate manner during muscle biogenesis.

Muscle cell differentiation involves significant gene reprogramming as well as cellular reshaping. The role and modulation of autophagy in muscle in several physiological and pathological conditions, including fasting, atrophy and exercise, have been deeply investigated (Vainshtein et al., 2014), but the functional relationship between autophagy and cell metabolism during muscle differentiation remains largely obscure.

Here we illustrate the main findings reported in our recently published paper (Fortini et al., 2016) where we exploited the ability of mouse skeletal Muscle Satellite Cells (MSC)-derived myoblasts to differentiate into myotubes to study the integrated network that cross-regulates autophagy metabolism reprogramming during myogenesis. The forkhead box O3 (FoxO3) transcription factor FoxO3, which is induced by oxidative stress (Li et al., 2015) and in atrophic skeletal muscle (Mammucari et al., 2007), is known to control the transcription of autophagy-related genes, including LC3. Consistently, we found that the mRNA level of FoxO3 and of LC3 increased up to three- and four-folds, respectively, during the transition from myoblast to myotube. Western blotting and immunofluorescence confirmed that autophagy was up-regulated soon after the MSC myoblasts were induced to differentiate into myocytes, and remained up-regulated during the fusion process leading to myotubes. Yet, the net production of autophagosomes slightly decreased in fully differentiated myotubes. Up-regulation of autophagy during the differentiation of myoblasts through the formation of mature myotubes has been also reported by Gottlieb and associates (Sin et al., 2016).

Macromolecular turnover is a requisite of the myogenic program. Consistent with this view, autophagy was not up-regulated in myocytes that were cultured at very low density, a condition that does not allow their fusion into myotubes. To further confirm the important role of autophagy in the myogenesis, we used two different approaches to prevent the induction of autophagy in MSC myoblasts, namely the presence of the antioxidant N-acetyl cysteine (NAC) during differentiation and the post-transcriptional silencing of Beclin 1. NAC has been shown to significantly decrease the basal autophagic flux in skeletal muscles of mice by limiting the production of reactive oxygen species (Rahman et al., 2014). Both these treatments effectively hampered the up-regulation of autophagy, and concomitantly we observed a remarkable reduction of the fusion index (by approximately two-folds).

mTORC1, a negative regulator of autophagy, plays a pivotal role in muscle biogenesis, as it controls multiple stages of the myofiber formation process (Erbay et al., 2003; Sun et al., 2010). Remarkably, mTORC1 remained active during the whole process of differentiation up to the myotube formation, and in spite of this autophagy was induced. Since the AMPk pathway was concomitantly induced along the myogenesis process, we speculate that AMPk overrides the mTOR inhibitory action gradually with time so that autophagy was modulated in a fashion compatible with the differentiation and fusion processes.

The inhibition of mTORC1 by rapamycin led to a further stimulation of autophagy, indicating that mTORC1 exerts a tonic inhibition on autophagy during the process. When the myoblasts were treated with rapamycin myotube formation was drastically impaired. The picture that emerges is that autophagy must be finely tuned and balanced in coordination with cell metabolism in order to allow the correct development of the skeletal muscle tissue.

The metabolic reprogramming during myogenesis was therefore studied in the same cell system. Interestingly, the transcription of PGC-1α, a master regulator of mitochondrial biogenesis, but not of PGC1α, was significantly up-regulated during muscle differentiation, and in parallel the number of mitochondrial DNA molecules as well as the levels of mitochondrial proteins also increased. The production of ATP+ADP, but not that of lactate, also increased during the myogenic process. These data are supportive of an increased mitochondrial activity in the passage from undifferentiated myoblasts to mature myotube. This is consistent with the recent findings reported by Sin et al. (2016) who additionally showed that a dynamic remodeling of the mitochondrial network, including mitochondrial clearance and biogenesis, is required during the myoblast/myotube transition (Figure 1). This mitochondrial remodeling was impaired when autophagy was blocked (Sin et al., 2016).

**Figure 1:**
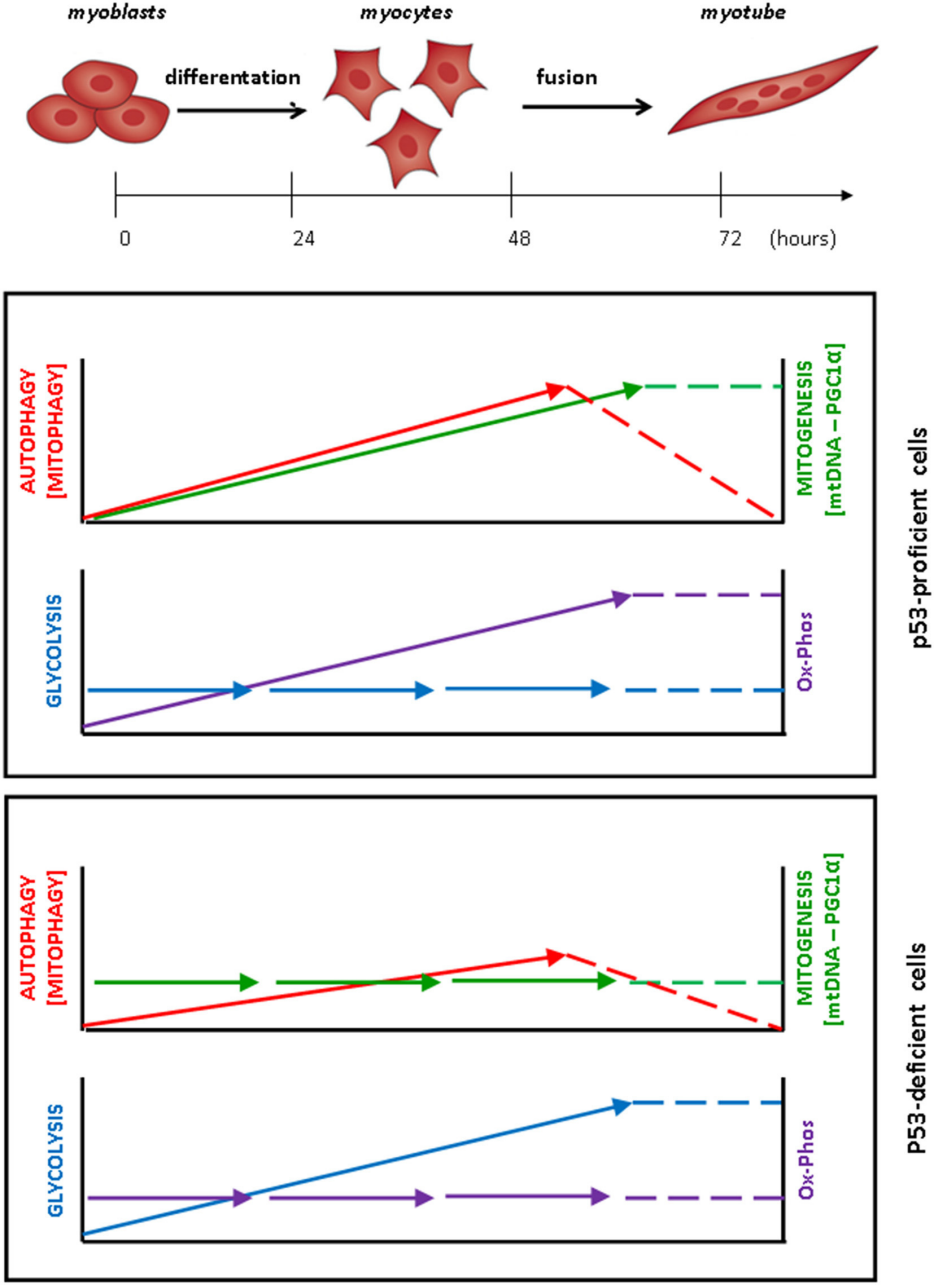
**Coordinated metabolic changes and modulation of autophagy during myoblasts differentiation and fusion into myotubes**. In p53-proficient MSC-derived myoblasts, autophagy and mitophagy are activated, increase along with the differentiation of myoblasts into myocytes and continue to increase up to the step of cell-to-cell fusion. Once the myotubes have formed, autophagy and mitophagy gradually slow-down to the basal level. The remodeling of the mitochondrial asset is testified by the constant increase in the content of mitochondrial DNA and protein (associated with increased expression of the transcription factor PGC1-α) and is paralleled by the increase in the oxidative phosphorylation (Ox-Phos), while the level of glycolysis is unchanged. In p53-deficient myoblasts, by contrast, the modulation of autophagy is greatly attenuated during the myogenesis process, mitogenesis is not induced and aggregates of abnormal mitochondria accumulate in the cell. In these cells, the production of lactate and ATP increases during the differentiation of myoblasts up to the formation of myotubes, indicating that their energetic metabolism relies essentially on glycolysis.

To get a more in depth insight into the mechanistic relationship between cell metabolism reprogramming, mitochondrial homeostasis and autophagy in the myogenic process, we performed a similar study in p53-null MSC-derived myoblasts cultivated under differentiation permissive conditions. In fact, p53 is known to affect myoblast differentiation (Porrello et al., 2000; Cam et al., 2006) and muscle metabolism (Park et al., 2009; Saleem et al., 2014), as well as autophagy (Maiuri et al., 2010; Rufini et al., 2013). Consistent with previous findings (Porrello et al., 2000; Fortini et al., 2012), the formation of myotubes was impaired in p53-null myoblasts, and in parallel we found that the induction of autophagy during the incubation in the differentiation condition was attenuated. Indeed, p53-null myoblasts showed abnormalities in the lysosomal apparatus that reflected in the reduced formation of autolysosomes. In contrast to what observed in p53-proficient myoblasts, in which the glycolytic rate did not change during myogenesis, in p53-null myoblasts an increased glycolytic flux occurred during their differentiation, as testified by the concurrent increase in lactate and in ATP+ADP. Further, in p53-null myoblasts, the mRNA level of PGC-1α remained unchanged, and the content of mtDNA, as well as the protein levels of COXIV and OXPHOS, did not increase in the course of differentiation. It is of note that p53 KO mice show greater fatigability and less locomotory endurance than wild-type animals, and this is associated with reduced expression of PGC-1α and diminished mitochondrial content and functionality in the gastrocnemius (Saleem et al., 2014) providing clear evidence that mitochondrial biogenesis and muscle performance are causally associated. We hypothesize that imperfect myogenesis in these cells could arise from impaired mitophagy. In fact, we observed that abnormal mitochondria forming aggregates accumulated in p53 null myoblasts.

In conclusion, we (Fortini et al., 2016) and others (Sin et al., 2016) demonstrated that the correct execution of the myogenesis program requires the concurrent and coordinated modulation of autophagy, cell metabolism, and mitochondrial remodeling. Further, we demonstrated that the lack of p53 attenuated autophagy and mitogenesis, caused the switch from aerobic respiration to glycolysis, and impaired myogenesis, thus highlighting the role of physiological p53 activity for muscle homeostasis (Fortini et al., 2016). The link between p53, oxidative stress, FoxO3, autophagy and mitogenesis during myogenesis clearly deserves a more in depth analysis. In this respect, it has been shown that, under starvation, FoxO activation, promotes the synthesis of glutamine, which in turn prevents the activation of mTOR and stimulates the autophagic flux (Van Der Vos et al., 2012). On the other hand, p53 has been shown to enhance mitochondrial respiration and ATP generation and to increase the cellular level of anti-oxidant GSH by promoting mitochondrial glutaminolysis (Hu et al., 2010).

A better understanding of the gene networks operating during muscle differentiation may open new avenues for the therapies of muscle disorders and repair.

## Author contributions

The manuscript was written by ED and CI; revised by PF and EI, read and approved by all co-authors.

### Conflict of interest statement

The authors declare that the research was conducted in the absence of any commercial or financial relationships that could be construed as a potential conflict of interest.
